# Accuracy of cup placement compared with preoperative surgeon targets in primary total hip arthroplasty using standard instrumentation and techniques: a global, multicenter study

**DOI:** 10.1186/s10195-024-00766-2

**Published:** 2024-05-10

**Authors:** Geert Meermans, David Fawley, Luigi Zagra, René H. M. ten Broeke, Kory Johnson, Thierry Bernard, Henry Clayton Thomason

**Affiliations:** 1 Department of Orthopaedics, Bravis Hospital, Bergen op Zoom, Roosendaal, The Netherlands; 2 DePuy Synthes, 700 Orthopaedic Drive, Warsaw, IN USA; 3 Hip Department, IRCCS Istituto Ortopedico Galeazzi, Milan, Italy; 4 Department of Orthopaedic Surgery, Maastricht University Medical Centre, 6202 AZ Maastricht, The Netherlands; 5 Orthopaedic Associates of Michigan, 555 Mid Towne St Suite 105, Grand Rapids, MI USA; 6 Carolina Orthopaedic & Sports Medicine Center, 2345 Court Dr., Gastonia, NC USA

**Keywords:** Total hip arthroplasty, Cup positioning, Cup angle, Version, Inclination, Abduction

## Abstract

**Background:**

Acetabular cup positioning in total hip arthroplasty (THA) is closely related to outcomes. The literature has suggested cup parameters defined by the Lewinnek safe zone; however, the validity of such measures is in question. Several studies have raised concerns about the benefits of using the Lewinnek safe zone as a predictor of success. In this study we elected to use prospective surgeon targets as the basis for comparison to see how successful surgeons are positioning their cup using standard instruments and techniques.

**Methods:**

A prospective, global, multicenter study was conducted. Cup positioning success was defined as a composite endpoint. Both cup inclination and version needed to be within 10° of the surgeon target to be considered a success. Radiographic analysis was conducted by a third-party reviewer.

**Results:**

In 170 subjects, inclination, target versus actual, was 44.8° [standard deviation (SD 0.9°)] and 43.1° (SD 7.6°), respectively (*p* = 0.0029). Inclination was considered successful in 84.1% of cases. Mean version, target versus actual, was 19.4° (SD 3.9°) and 27.2° (SD 5.6°), respectively (*p* < 0.0001). Version was considered successful in 63.4% of cases, and combined position (inclination and version) was considered successful in 53.1%.

**Conclusion:**

This study shows that with traditional methods of placing the cup intraoperatively, surgeons are only accurate 53.1% of the time compared with a predicted preoperative plan. This study suggests that the inconsistency in cup positioning based on the surgeon’s planned target is potentially another important variable to consider while using a mechanical guide or in freehand techniques for cup placement in THA.

*Trial Registration*: This study is registered on ClinicalTrials.gov, NCT03189303.

## Introduction

Acetabular cup positioning in total hip arthroplasty (THA) has been shown to be closely related to both short- and long-term outcomes. Malposition has been linked to higher rates of dislocation [1–3], impingement of the prosthetic neck against the acetabular rim [4], bearing surface wear [5–15], squeaking and ceramic breakage [16, 17], poor biomechanics [18, 19], leg length discrepancy [20], groin pain and reduced range of motion [21–23], pelvic osteolysis [3], and revision. Some studies have also demonstrated an increased risk of malposition with low-volume surgeons [23], higher patient body mass index (BMI) [23, 24], and minimally invasive surgical technique [25], although these findings have not been corroborated by other studies [23, 26–28]. Cup positioning success is often assessed in relation to the “safe zones” described by Lewinnek [29], who defined a range of 30–50° of inclination (abduction) and 5–25° of anteversion for minimizing dislocation risk. There are a number of investigations that cast doubt on this zone being adequate to predict cup positioning success for all patients and all approaches, since dislocation risk is comparable with placement outside of this “safe zone” [30–33]. There is also a growing body of evidence to support the need for unique strategies to combat spinopelvic issues [34, 35]. A surgeon should be able to place the cup in the orientation that best fits the needs of the patient [36] according to intraoperative landmarks [37]. As we develop a “true safe zone” it is critical that we understand how accurately we can achieve cup placement given a specific target and in this study we define success as the ability of the surgeon to orient the cup within 10° of their established target for radiographic inclination and version [38]. Secondarily, we collected short-term clinical outcomes and complications to allow for future study of any relevant correlations to cup position.

## Materials and methods

A global, prospective, multicenter study was conducted at eight sites between August 2017 and December 2021. One experienced medium-volume (50–100 primary THA cases per year) surgeon performed all procedures at each center. Informed consent was collected for all subjects prior to participation. Preoperative assessments included Harris Hip Score [39], EQ-5D-5L [40, 41], radiographs [anteroposterior (AP) hip, AP pelvis, lateral], baseline patient reported early functional recovery outcomes, and surgeon preoperative planning details (target cup inclination and version angles and cup size based on surgeon’s preference). A Pinnacle acetabular cup (DePuy Synthes, Warsaw, IN, USA) was used in all cases. Surgeries were performed via posterior or anterolateral approach with the subject in lateral decubitus position. No imaging or navigation was used to aid in placement of the acetabular components. Surgeons could place the cup freehand or with a mechanical guide. Intraoperative complications were recorded, along with operative datapoints of primary diagnosis, ASA risk, surgery duration, surgical approach, incision measurement, screw use, bone class, and osteophyte removal. Date of discharge and discharge disposition was recorded. Postoperative assessments included Harris Hip Score, EQ-5D-5L, Forgotten Joint Score (FJS-12) [42], radiographs (AP hip, AP pelvis, lateral), and patient reported early functional recovery outcomes. Complications (all serious and device- or procedure-related) were recorded from the time of subject consent to end of study participation.

### Statistical analysis

This study was not powered to detect a specific difference in the primary endpoint, instead the sample size was estimated to allow a certain margin of error in the proportion of acetabular cups successfully positioned as measured from postoperative radiographs. Cup positioning success was defined as a composite endpoint. Both cup inclination and cup version needed to be within 10° of the surgeon target to be considered a success. The number of subjects and the proportion with acetabular cup position success and its two-sided 95% confidence intervals were evaluated using the binomial exact method. Data from the films collected during the 6-week visit were used whenever available for primary endpoint analysis. If the films necessary for primary endpoint analysis were missing, incomplete or of poor quality, the films collected at the 12-week visit were used. Patient-reported outcomes scores were summarized with descriptive statistics.

### Radiographic analysis

Acetabular cup inclination and version was measured as described by Wan et al. [43]. Inclination was defined as the angle between the face of the acetabular shell and the transverse axis of the subject via obturator foramen or pelvic tear drops, and version as the angle between the acetabular axis and the coronal plane; both were measured from standing AP pelvis radiographs. Additionally, if present, migration/subsidence, radiolucency, osteolysis, fracture, sclerotic lines, and heterotopic ossification were documented and summarized (femoral and acetabular). The AP standing radiographs had to comply with the following criteria: distance between the symphysis and the sacrococcygeal joint of ~ 30mm (10–40 mm) in men and 50 mm (40–60 mm) in women to exclude abnormal pelvic tilt in the sagittal plane [44, 45] and the coccyx centered on the pubic symphysis to exclude rotation of the pelvis in the transverse plane [45]. In the absence of a full lower extremity long standing AP X-ray view, proximal femur/pelvis vertical alignment was estimated from the weight-bearing AP pelvis X-ray view. Proximal femur/pelvis vertical alignment was determined by measuring the difference of the vertical distances between the left and right pelvic teardrops and the center of the respective femoral heads to provide an estimate of leg-length discrepancy [46]. All radiographic analysis was conducted by a single, independent, third-party, practicing board-certified musculoskeletal radiologist reviewer (Medical Metrics, Inc., Houston, TX, USA).

### Patient demographics

A total of 184 subjects were enrolled in the study, 171 were treated. One subject was treated via anterior approach and was excluded from analysis, leaving a total of 170 in the per-protocol analysis set. Mean age was 65.8 (SD 8.9) years, mean BMI was 29.7 (SD 6.1), and 108 (63.5%) hips were women. Primary diagnosis was osteoarthritis in 150 (88.2%) hips. Acetabular bone class was reported as normal or good in 152 (89.4%) hips. Osteophytes were removed in 83 (48.8%) cases. Acetabular screws were used in 27 (15.9%) cases. Additional demographic and surgical details are presented in Table 1.

**Table 1:** Patient demographics and surgical details

	Mean	SD	Range	*n*	
Age (years)	65.8	8.9	35–85	170	
BMI (kg/m^2^)	29.7	6.1	17.4–54.6	170	
Skin-to-skin time (min)	62.0	18.0	33–119	170	
Incision measurement (cm)	14.6	3.0	8–21	167	
Length of stay (days)	2.1	1.6	0–7	170	
Gender					
ASA risk	I 17 (10.0%)	II 113 (66.5%)	III 39 (22.9%)	IV 1 (0.6%)	
Primary diagnosis	OA 150 (88.2%)	AVN 8 (4.7%)	CDH/DDH 7 (4.1%)	Other 5 (2.9%)	
Operative side	Left 79 (46.5%)	Right 91 (53.5%)			
Bone class	Normal 75 (44.1%)	Good 77 (45.3%)	Fair 9 (5.3%)	Poor 2 (1.2%)	Sclerotic 7 (4.1%)
Surgical approach	Posterior 108 (63.5%)		Anterolateral (modified hardinge) 62 (36.5%)		
Discharge disposition	Home 115 (67.6%)	Home health care 37 (21.8%)		Short-term rehab facility 18 (10.6%)	

## Results

One subject died due to an accident unrelated to their hip surgery. Eight intraoperative fractures were reported: three greater trochanter, three acetabular, one pubic rami, and one femoral. The other serious or hip-related complications reported were dislocation (two; one recurrent), wound secretion and infection treated with revision one day after the initial surgery (one), wound infection (one), acetabular loosening (one), lower extremity numbness (one), foot drop (one), renal insufficiency (one), and urosepsis (one).

Clinical outcomes are outlined in Table 2. The Harris hip score improved from a mean of 51.3 preoperatively to 92.7, 12 weeks postoperatively. The EQ-5D-5L score improved from a mean of 0.59 preoperatively to 0.85, 12 weeks postoperatively. The EQ-5D-VAS score improved from a mean of 67.9 preoperatively to 83.6, 12 weeks postoperatively. Patients stated they were “extremely” or “very” satisfied postsurgery, a combined 93.2% of the time. We reviewed any potential correlations between clinical outcomes and cup position but there were no significant differences between groups.

**Table 2:** Clinical outcomes

	Mean	SD	*n*	*p* value^a^	
Harris hip total score (range 0–100)					
Preoperative	51.3	17.1	141	< 0.0001	
6 weeks (14–60 days)^b^	84.2	14.2	124	< 0.0001	
12 weeks (61–180 days)	92.7	9.7	143		
EQ-5D-5L					
Preoperative	0.59	0.18	169	< 0.0001	
6 weeks (14–60 days)	0.79	0.13	151	< 0.0001	
12 weeks (61–180 days)	0.85	0.14	157		
EQ-5D VAS (range 0–100)					
Preoperative	67.9	19.1	169	< 0.0001	
6 weeks (14–60 days)	80.9	14.1	151	< 0.0001	
12 weeks (61–180 days)	83.6	13.6	158		
Forgotten joint score (range 0–100)					
Preoperative	–	–	–	< 0.0001	
6 weeks (14–60 days)	46.0	29.0	151		
12 weeks (61–180 days)	63.4	28.3	158		

	None	Mild	Moderate	Severe	
Buttock pain [*n* (%)]					
Preoperative	34 (20.1)	42 (24.9)	65 (38.5)	28 (16.6)	
6 weeks (14–60 days)	87 (51.2)	45 (26.5)	17 (10.0)	2 (1.2)	
12 weeks (61–180 days)	103 (60.6)	41 (24.1)	13 (7.6)	0 (0.0)	
Groin pain [*n* (%)]					
Preoperative	23 (13.6)	21 (12.4)	78 (46.2)	47 (27.8)	
6 weeks (14–60 days)	77 (45.3)	50 (29.4)	20 (11.8)	4 (2.4)	
12 weeks (61–180 days)	110 (64.7)	36 (21.2)	10 (5.9)	2 (1.2)	

	Extremely satisfied	Very satisfied	Moderately satisfied	Slightly satisfied	Not at all satisfied
Patient satisfaction [*n* (%)]					
Preoperative^c^	64 (37.6)	95 (55.9)	7 (4.1)	0 (0.0)	3 (1.8)
6 weeks (14–60 days)	82 (48.2)	60 (35.3)	8 (4.7)	1 (0.6)	0 (0.0)
12 weeks (61–180 days)	93 (54.7)	53 (31.2)	11 (6.5)	1 (0.6)	0 (0.0)

	Equal	Right longer	Left longer		
Patient perception of leg length [*n* (%)]					
Preoperative	118 (71.5)	115 (67.6)	130 (76.5)		
6 weeks (14–60 days)	24 (14.5)	19 (11.2)	13 (7.6)		
12 weeks (61–180 days)	23 (13.9)	12 (7.1)	11 (6.5)		

^a^Change from baseline

^b^ROM was not collected at the 6-week visit for some subjects, per surgeon discretion

^c^Preop is patient expectation of satisfaction

**Fig. 1:**
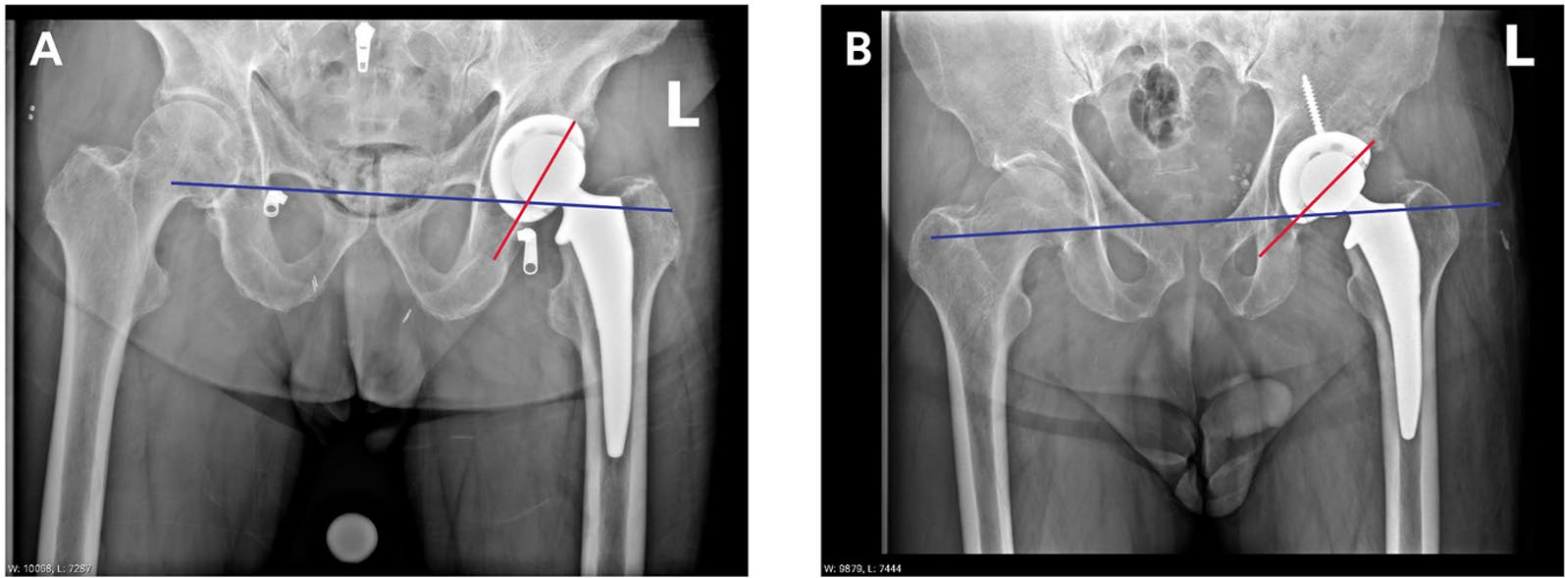
AP pelvis radiographs from two study cases at 6 weeks postoperatively. **A** Radiographic inclination of 62.8°; anteversion of 32.5°. **B** Radiographic Inclination of 42.0°; anteversion of 21.6°

Cup positioning outcomes are outlined in Table 3; radiographic outcomes in Table 4. The mean inclination at the first available postoperative visit, target versus actual, was 44.8° (standard deviation 0.9°) and 43.1° (standard deviation 7.6°), respectively (*p* = 0.0029). The mean difference was −1.7°. A total of 138 of the 164 (84.1%) patients measured had a “successful” cup position with regards to abduction, or inclination, being within 10° of the stated target. The mean version at the first available postoperative visit, target versus actual, was 19.5° (standard deviation 3.9°) and 27.2° (standard deviation 5.6°), respectively (*p* < 0.0001). The mean difference was 7.8°. A total of 102 of the 161 (63.4%) patients measured had a “successful” cup position with regards to version, being within 10° of the stated target (Fig. 1).

**Table 3:** Cup positioning outcomes (first available visit)

	Target (SD; *N*)	Actual-radiographic (SD; *N*)	*p* value	Mean target difference (SD; *N*)	Success rate
Inclination	44.8° (0.9°; 170)	43.1° (8.0°; 164)	0.0029	−1.7° (7.4°; 164)	84.1% (138 of 164)
Anteversion	19.5° (3.9°; 170)	27.2° (5.6°; 161)	< 0.0001	7.8° (6.0°; 161)	63.4% (102 of 161)
Combined					53.1% (86 of 162)

**Table 4:** Radiographic outcomes

Week 12 (*n* = 157)				
Acetabular radiolucency present	8.3%			
Acetabular osteolysis present	None			
Acetabular sclerotic lines present	1.3%			
Acetabular migration present	None (*n* = 69)			
Femoral stem radiolucency present	0.6%			
Femoral stem osteolysis present	None			
Femoral stem sclerotic lines	6.4%			
Femoral subsidence present	None			
Femoral stem position	Neutral 75.8%	Valgus 1.9%	Varus 21.7%	
Heterotopic ossification	Class 0 70.7%	Class I 26.1%	Class II 1.9%	Class III 1.3%
Proximal femur/pelvis vertical alignment (mm)	Mean 4.2	SD 3.7

**Fig. 2:**
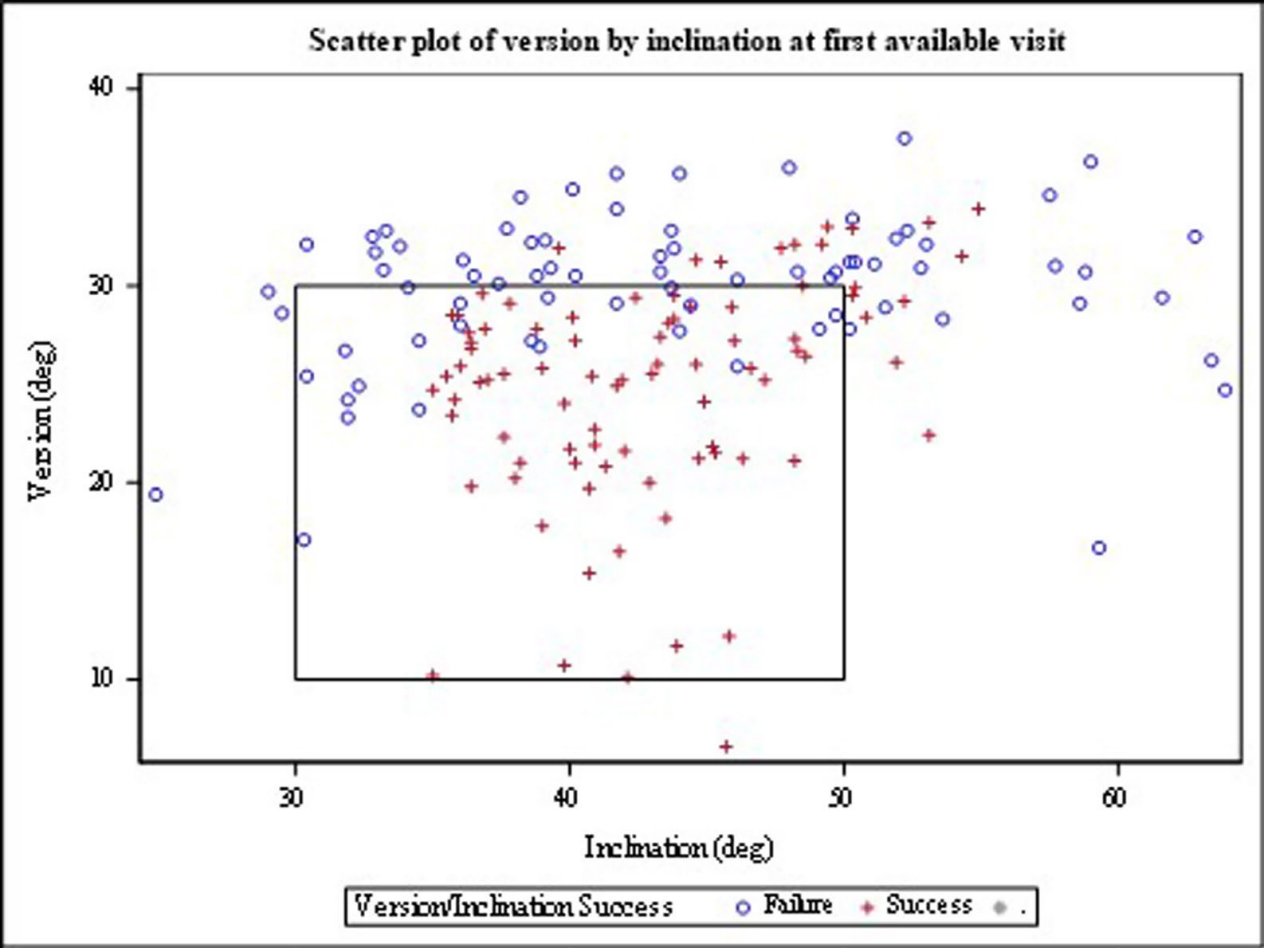
This graph shows the inclination (horizontal axis) and anteversion angles (vertical axis) for the study population at first available postoperative visit. The box indicates the Lewinnek safe zone (only for reader reference, as the safe zone was not used for measuring success in this evaluation)

**Fig. 3:**
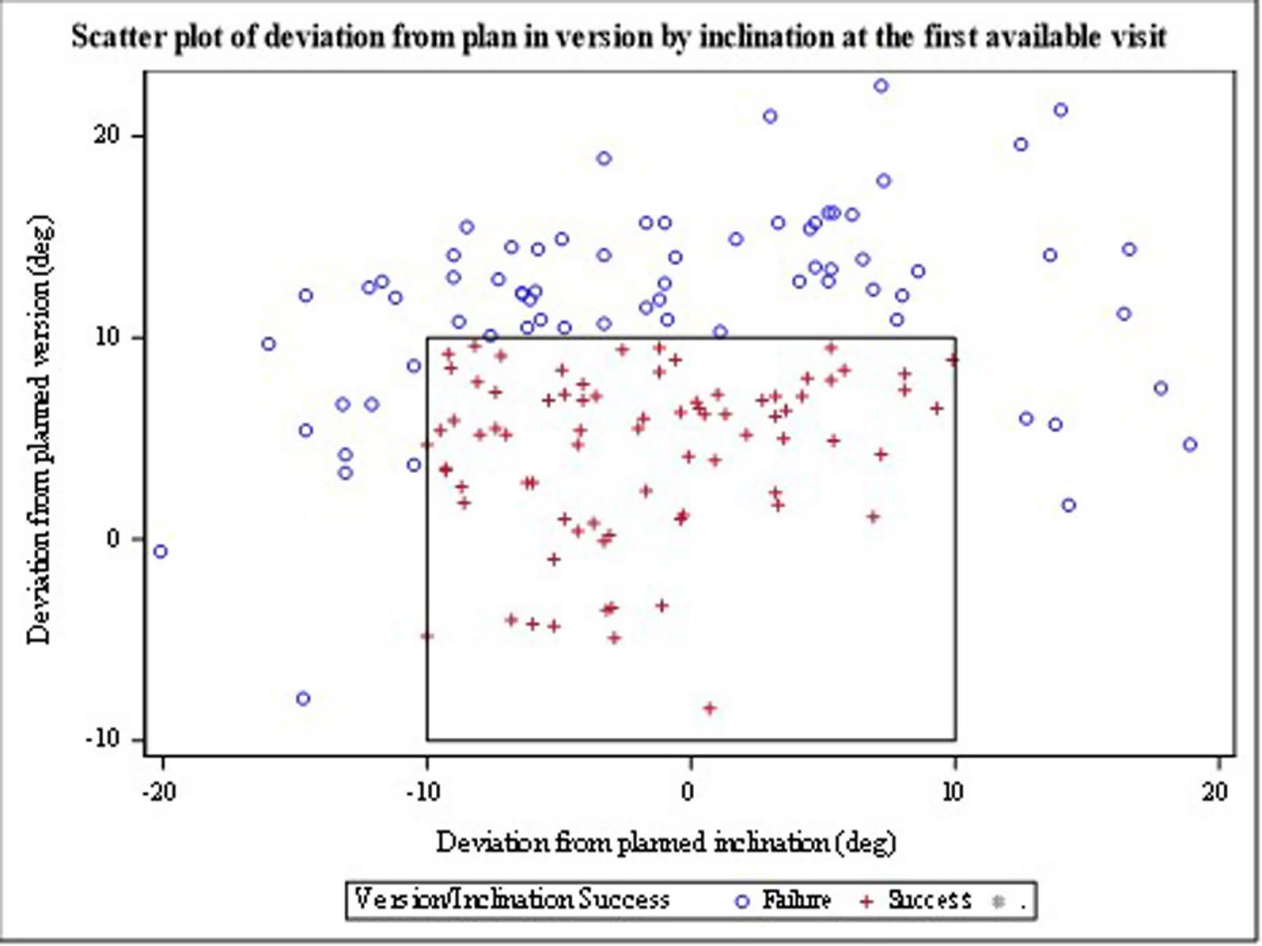
This graph shows the difference from surgeon target for inclination (horizontal axis) and version angles (vertical axis) for the study population at first available postoperative visit

For total cup positioning, 86 of 162 (53.1%) patients had a “successful” cup position with regards to inclination and version being within 10° plus or minus of the stated targets. Figure 2 is a scatter plot of the results of this study superimposed on Lewinnek’s “safe zone” for reference. Figure 3 shows the difference between the surgeon targets and the measured radiographic inclination and anteversion angles. We stratified cup positioning results by surgical approach and found that inclination and overall cup positioning varied between groups. Inclination was successful in 92.7% of anterolateral cases compared with 71.9% of posterior cases. There were not significant differences for version success or overall success.

## Discussion

In this prospective, global, multicenter study, we compared the radiographic inclination and version angles of the cup with the pre-/intraoperatively defined target by the surgeon. We found that the mean radiographic inclination of the cup was slightly lower than targeted for with 84% within the desired range of ± 10°. The mean radiographic anteversion was significantly higher than targeted with 63% within the desired range of ± 10°. For the combined radiographic inclination and version, only 53% were within the desired zone. These data demonstrate that with traditional methods, surgeons are only able to implant a cup in a targeted orientation in slightly more than half of their patients.

Many studies have shown that a successful cup position by radiographic criteria and a perceived “safe zone” does not mean a successful outcome [47], and others have shown that a cup position out of the same “safe zone” does not mean failed outcomes [48]. Clearly the outcomes and possible complications, such as dislocation, are multifactorial and involve surgical approach, combined version (the cup and the femoral stem versions combined), pelvic tilt and obliquity, and spinal conditions. Table 5 shows the success rates of similar studies [23, 25, 27, 49–53]. As far as the authors are aware, this is the first prospective study conducted to review cup positioning success in THA as previous studies have utilized retrospective radiographic review to the Lewinnek safe zone instead of the prospective preoperative target.

**Table 5:** Results of freehand cup placement in the literature

Authors	Anteversion	Abduction	Inside target zone (%)
Barrack et al. [23]	5–35°	30–55°	88
Bosker et al. [49]	5–25°	30–50°	70.5
Callanan et al. [25]	5–25°	30–45°	47
DiGioia et al. [50]	5–25°	30–50°	20.3
Hassan et al. [51]	5–25°	30–50°	58
Leichtle et al. [52]	10–30°	35–55°	65.5
Reize et al. [27]	5–25°	30–50°	41
Saxler et al. [53]	5–25°	30–50°	25.7

The current study was not designed to determine a “safe zone” for acetabular cup position to predict stability and outcomes as many others have. The factors that constitute successful outcomes and prevent complications are multifactorial and should be individualized by each surgeon for each patient. Several methods have been described for improving the accuracy of implant positioning in everyday clinical practice, including intraoperative landmarks that can be safely used to control the orientation of the cup, such as the transverse acetabular ligament [37]. Increasing knowledge of spino-pelvic issues advocates tailored implant positioning for each patient with specific consideration to cup orientation. This study was designed to evaluate how accurate the cup position was compared to the surgeon target and the preoperative plan, using plain film radiographs.

Since mean BMI in our population was nearly 30, we reviewed cup positioning success by BMI category [less than 18.5 (underweight), 18.5–24.9 (normal), 25.0–29.9 (overweight), and 30.0 and over (obese)]. We found that cup positioning success increased with BMI. Overall cup positioning success was 42% for normal BMI, 46% for overweight, and 63% for obese. However, due to the relatively low numbers in these groups (36, 54, and 71, respectively), these results should be evaluated with caution.

Limitations of this study first include the fact that it is not a randomized study, but a prospective cohort study. Moreover, the study is multicenter, so different surgical landmarks, surgical approaches, and techniques have been used by these surgeons. However, all the procedures were performed in lateral decubitus position, without any imaging or navigation support and the comparison were not among different surgical techniques, but with regards to surgeons’ targets, thus depicting a common scenario of clinical practice by experienced surgeons with similar annual primary THA volumes. Also acknowledged is the fact that interpreting radiographs is an inherently subjective process. We attempted to mitigate this difference by using one third party reader for all radiographs. Making a measurement of a 3D position from a 2D radiograph is also inherently flawed but is what we have used as a historical standard. According to Wan et al. [43], we defined radiographic inclination and anteversion based on the coronal plane of the patient, as seen on the anteroposterior pelvic radiograph, but pelvic tilt and rotation have been shown to alter appearances greatly on radiographs, accounting for varied readings of angles and measurements. As this is a multicenter study across continents with many different radiology technicians taking films, patient position was likely not uniform, despite training all for standardization.

As a result of trying to interpret and place the cup as accurately as possible, newer techniques have started to become more popular such as using live fluoroscopy in the operating room, as well as using computer navigation. Both techniques are not without their own issues though, including a learning curve for use, increasing time in the operating room, issues for surgeons confident in operating on lateral decubitus, and adding a substantial cost to the procedure.

## Conclusion

Acetabular cup position is a critical factor in THA. This study shows that with traditional methods of placing the cup intraoperatively, and by using traditional radiographic interpretation postoperatively, surgeons are only accurate 53.1% of the time with regard to both inclination and version, compared with a predicted preoperative plan. As more factors continue to become apparently increasingly important for patient outcomes, such as spinopelvic characteristics, the accuracy of cup placement will become more crucial. This study suggests that incorporating techniques to improve accuracy in the placement of the cup is also important to consider.

## Data Availability

Data will be made available on reasonable request.
